# Incidence and Risk Factors of Hypomagnesemia in Head and Neck Cancer Patients Treated with Cetuximab

**DOI:** 10.3389/fonc.2016.00196

**Published:** 2016-09-14

**Authors:** Tomohiro Enokida, Shinya Suzuki, Tetsuro Wakasugi, Tomoko Yamazaki, Susumu Okano, Makoto Tahara

**Affiliations:** ^1^Department of Head and Neck Medical Oncology, National Cancer Center Hospital East, Kashiwa, Japan; ^2^Division of Pharmacy, National Cancer Center Hospital East, Kashiwa, Japan

**Keywords:** hypomagnesemia, cetuximab, head and neck cancer, squamous cell carcinoma, chemotherapy, radiotherapy

## Abstract

**Background:**

Hypomagnesemia is a common adverse event during cetuximab (Cmab) treatment. However, few reports have investigated the incidence and risk factors of hypomagnesemia in head and neck cancer patients treated with Cmab.

**Methods:**

We retrospectively reviewed 131 head and neck cancer patients who received Cmab-containing therapy. Main eligibility criteria were ≥3 Cmab administrations, no prior EGFR-directed therapy, and no prophylactic Mg supplementation.

**Results:**

Median baseline serum Mg level and number of Cmab administrations were 2.2 mg/dl and 8, respectively. Overall incidence of hypomagnesemia was 50.4% (grade 1, 46.6%; grade 2, 3.1%; grade 3, 0%; and grade 4, 0.8%) and differed between patients treated with palliative chemotherapy and bioradiation (Cmab and radiation) (63 versus 24%; *P* < 0.01). Independent risk factors were low baseline serum Mg [odds ratio (OR) 161.988, 95% confidence interval (CI) 9.436–2780.895], ≥7 Cmab administrations (OR 3.56, 95% CI 1.16–13.98), and concurrent administration of platinum (cisplatin; OR 23.695, 95% CI 5.219–107.574, carboplatin; OR 5.487, 95% CI 1.831–16.439). Respective incidence of hypomagnesemia in patients in high- (concurrent platinum and ≥7 Cmab administrations) and low-risk (no concurrent platinum and <7 Cmab administrations) groups was 66.0 and 6.6% (*P* < 0.001, OR 28.0).

**Conclusion:**

Cmab is associated with a significant risk of hypomagnesemia in patients with head and neck cancer with longer term administration and concurrent platinum therapy. High-risk patients should be treated with particular care.

## Introduction

Cetuximab (Cmab) is a human–murine monoclonal antibody directed against the EGFR protein and is the only approved molecular targeted drug for the treatment of squamous cell carcinoma of the head and neck. Cmab has been demonstrated to enhance sensitivity to radiotherapy and chemotherapy and improve overall survival (1, 2). As a single agent, Cmab has a response rate of 13% in patients with platinum-refractory head and neck squamous cell carcinoma (3). This background will likely increase the use of this agent and in turn increase the incidence of toxicities associated with the prolonged use of Cmab.

The use of anti-EGFR mAbs is associated with a number of unique adverse events. Previous meta-analyses have shown an increased risk of rash, nail changes, and venous thromboembolism (4–6). Hypomagnesemia associated with Cmab has also been reported. Hypomagnesemia can result in cardiac arrhythmia, coronary artery vasospasm, and sudden cardiac death, and is a serious adverse event in patients treated with Cmab. However, the symptoms of hypomagnesemia may be fairly non-specific, including irritability, paresthesia, and severe fatigue, which are easily attributable to the underlying tumor or to previous chemotherapy regimens (7). Thus, the diagnosis of Cmab-induced hypomagnesemia may be overlooked, and the impact of the condition may be underestimated. Moreover, few studies that focused on the incidence and risk factors of hypomagnesemia in head and neck cancer patients treated with Cmab.

Here, we retrospectively reviewed the incidence and effects of hypomagnesemia in a series of head and neck cancer patients who received Cmab-containing therapy.

## Patients and Methods

We reviewed the medical records of patients treated with Cmab-containing treatment at the National Cancer Center Hospital East Japan between February 2012 and March 2014. Main eligibility criteria included age ≥20 years, no prior history of EGFR-directed therapy, ≥3 Cmab administrations, and no prophylactic Mg supplementation and stage III or IV advanced head and neck cancer. All patients received Cmab at the dose of 400 mg/m^2^ IV on day 1 and 250 mg/m^2^ weekly thereafter. In the Cmab plus radiation (bioradiation) group, Cmab was given for the duration of radiotherapy. Patients treated with palliative chemotherapy were eligible to receive a platinum agent (cisplatin, CDDP; or carboplatin, CBDCA). In these settings, patients who had at least stable disease received Cmab monotherapy until disease progression or until unacceptable toxic effects after a maximum of six cycles of platinum administration. Serum Mg levels were recorded before administration at least once every week during the treatment of Cmab. The study was approved by the Clinical Research and Ethical Review Board of our institutional hospital (task number: 2013-283).

### Statistical Analysis

The incidence of hypomagnesemia was calculated using the number of patients with hypomagnesemia and the total number of patients receiving Cmab treatment. The primary endpoint was the incidence of hypomagnesemia (i.e., Mg concentration below the lower limit of normal), and grade was recorded according to CTCAE version 4.0. The predictive value for hypomagnesemia was assessed by Cox regression models in multivariate analysis, with adjustment for the following potential prognostic variables: baseline serum Mg and calcium level, baseline creatinine clearance calculated from the Cockcroft–Gault formula (≥60 versus <60 ml/min), gender (male versus female), age (<60 versus ≥60 years), nutrition risk index calculated from serum albumin and body weight (8) (≥100 versus <100), number of Cmab administrations (<7 versus ≥7), concurrent platinum administration (absent versus present), history of platinum administration (absent versus present), and grade of rash (0–1 versus ≥2). SPSS version 21 software (SPSS Inc., Chicago, IL, USA) was used for all statistical analyses.

## Results

### Patient and Treatment Characteristics

A total of 131 patient cases were available for analysis. Most patients were men (79%) with a median age of 63 years (range 24–79 years). Main primary disease sites included the hypopharynx (24%) and oropharynx (20%). Eight patients (6%) had stage III disease and the remainder (94%) had stage IV. Over half of the patients had a history of platinum administration; among these, median time from the last administration of platinum to the initiation of Cmab was 154 days (range 7–2650). Median baseline serum Mg level, serum Ca level, and creatinine clearance were 2.2 mg/dl (range 1.8–2.6), 9.4 mg/dl (range 7.4–11.4), and 74.5 ml/min (range 29.7–195.0), respectively (Table 1). The most frequent treatment form was palliative chemotherapy (48%), and the median number of cycles of Cmab administration was 8 (range 3–65) (Table 2).

**Table 1:** **Patient and disease characteristics (*N* = 131)**.

Characteristic	
Median age (years) (range)	63 (24–79)
Sex, *n* (%)	
Male/female	103 (79)/28 (21)
Stage, *n* (%)	
III/IV	8 (6)/123 (94)
Primary site, *n* (%)	
Oral cavity	17 (13)
Oropharynx	26 (20)
Hypopharynx	32 (24)
Nasopharynx	21 (16)
Larynx	11 (8)
Nasal cavity/paranasal sinus	16 (12)
Salivary gland	6 (5)
Unknown primary site	1 (1)
Others	1 (1)
Initial median Mg level (mg/dl) (range)	2.2 (1.8–2.6)
Initial median adjusted Ca level (mg/dl) (range)	9.4 (7.4–11.4)
Initial median CCr^a^ (ml/min) (range)	74.5 (29.7–195.0)
Nutrition risk index^b^ (range)	104 (66–132)
Platinum history, *n* (%)	
Yes	48 (37)
No	83 (63)

*CCr, creatinine clearance*.

*^a^Cockcroft–Gault equation*.

*^b^1.519 × serum albumin + 41.7 × actual weight/ideal weight*.

**Table 2:** **Treatment characteristics (*N* = 131)**.

Characteristic	
Treatment form, *n* (%)	
Bioradiation	25 (19)
Induction chemotherapy	
PTX + CBDCA + Cmab	30 (23)
DTX + CDDP + Cmab	10 (8)
PTX + Cmab	1 (1)
CDDP + 5-FU + Cmab	1 (1)
Palliative chemotherapy	
PTX + CBDCA + Cmab	25 (19)
Cmab alone	16 (12)
CDDP + 5-FU + Cmab	16 (12)
PTX + Cmab	5 (4)
DTX + Cmab	1 (1)
CBDCA + 5-FU + Cmab	1 (1)
Median no. of Cmab administrations, *n* (range)	8 (3–60)
Concurrent platinum (%)	
Yes	83 (63)
No	48 (37)

*PTX, paclitaxel; CBDCA, carboplatin; Cmab, Cetuximab; DTX, docetaxel; CDDP, cisplatin; 5-FU, 5-fluorouracil*.

### Incidence and Risk Factors of Hypomagnesemia

**Figure 1:**
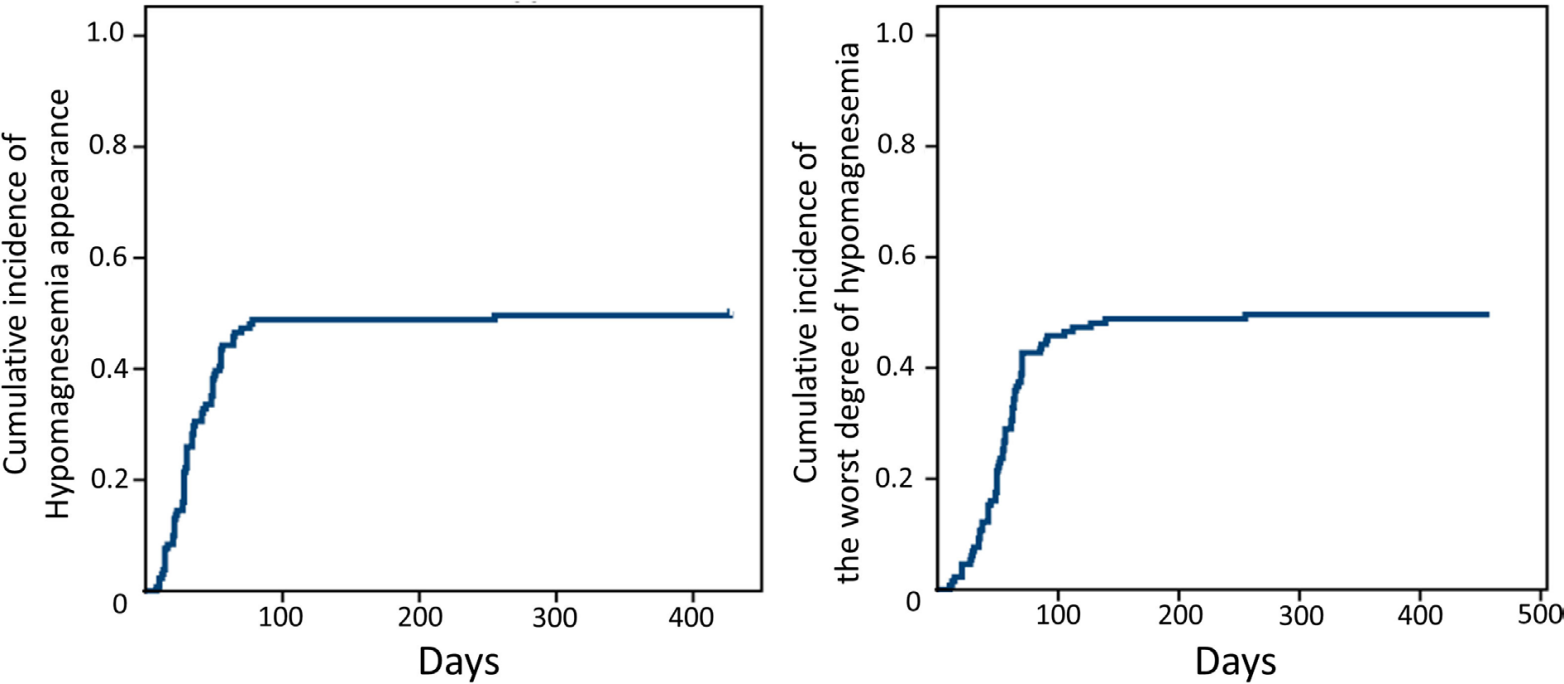
**Time to appearance (left) and the worst degree (right) of hypomagnesemia**.

Almost all patients (96%) had a decrease in serum Mg level during treatment compared with baseline measurements. The median Mg reduction was 0.4 mg/dl (range 0 to −1.2), which was corresponding to 20% (0–67). A higher (≥2.2 mg/dl) baseline serum Mg level was associated with a steeper slope (mean reduction: −0.51 versus −0.39 mg/dl; *P* = 0.012). The overall incidence of hypomagnesemia was 50.4% (grade 1, 46.6%; grade 2, 3.1%; grade 3, 0%; and grade 4, 0.8%), with a median follow up of 76 days (range 20–624). Median time to the onset of hypomagnesemia was 32 days (range 8–427), and the most severe decrease was seen at 55 days (range 10–455) (Figure 1). The incidence of hypomagnesemia varied according to treatment form, being higher in patients treated with palliative chemotherapy than in those treated with bioradiation (63 versus 24%; *P* < 0.001, Table 3). On multivariate analysis, low baseline serum Mg [odds ratio (OR) 161.988, 95% confidence interval (CI) 9.436–2780.895], ≥7 Cmab administrations (OR 3.556, 95% CI 1.16–13.98), and concurrent administration of CDDP (OR 23.695, 95% CI 5.219–107.574) or CBDCA (OR 5.487, 95% CI 1.831–16.439) were associated with hypomagnesemia (Table 4). Respective incidence of hypomagnesemia in patients in the high- (concurrent platinum and ≥7 Cmab administrations) and low-risk (no concurrent platinum and <7 Cmab administrations) groups was 66.0 and 6.6% (*P* < 0.001, OR 28.0; Table 5), respectively. Serum Ca level, which influences serum Mg level, was assessed in all patients at the time of the worst hypomagnesemia. The overall incidence of hypocalcemia was low (grade 1, 2%; grade 2, 2%), and a statistically significant correlation between these decreases during treatment was not seen (Spearman’s rho = 0.154, *P* = 0.104). The most commonly observed symptom considered to be related to hypomagnesemia was cramps (23%). No mental alteration or seizures were recorded.

**Table 3:** **Incidence of hypomagnesemia according to treatment form**.

Treatment form	*n* (%)
Bioradiation (*n* = 25)	6 (24)
Induction chemotherapy (*n* = 43)	20 (47)
Palliative chemotherapy (*n* = 63)	40 (63)

**Table 4:** **Multivariate analysis of hypomagnesemia**.

Variable	Odds ratio	95% CI	*P*-value
Initial Mg (mg/dl)	161.988	9.436–2780.895	<0.001
Initial Ca (mg/dl)	0.717	0.293–1.752	0.465
Initial CCr (ml/min)		0.748–8.726	0.134
≥60 (*n* = 104)	Referent		
<60 (*n* = 27)	2.556		
Age (year)		0.363–2.295	0.849
<60 (*n* = 54)	Referent		
≥60 (*n* = 77)	0.914		
Nutrition risk index		0.623–4.104	0.329
≥100 (*n* = 83)	Referent		
<100 (*n* = 48)	1.599		
Cmab cycle, *n*		1.16–13.98	0.02
<7 (*n* = 35)	Referent		
≥7 (*n* = 96)	3.556		
Concurrent CDDP		5.219–107.574	<0.001
No (*n* = 104)	Referent		
Yes (*n* = 27)	23.695		
Concurrent CBDCA		1.831–16.439	0.002
No (*n* = 71)	Referent		
Yes (*n* = 60)	5.487		
Platinum history		0.729–5.009	0.188
No (*n* = 80)	Referent		
Yes (*n* = 51)	1.911		
Rash (grade)		0.468–3.187	0.683
<2 (*n* = 89)	Referent		
≥2 (*n* = 42)	1.221		

*CCr, creatinine clearance*.

**Table 5:** **Serum Mg change according to risk classification**.

	High-risk group *n* = 66	Low-risk group *n* = 15	*P*-value	Odds ratio
	Cmab cycle ≥7 and concurrent platinum (+)	Cmab cycle <7 and concurrent platinum (−)		
Median initial Mg (range)	2.2 (1.8–2.6)	2.2 (1.8–2.6)	–	–
ΔMg (mg/dl)^c^ (range)	−0.5 (0 to −1.2)	−0.3 (0 to −0.8)	0.001^a^	–
ΔMg%^d^ (range)	−21 (0 to −67)	−13 (0 to −31)	0.001^a^	–
Hypomagnesemia^e^	44 (66.6%)	1 (6.6%)	<0.001^b^	28.0

*^a^Mann–Whitney U test*.

*^b^Fisher’s exact test*.

*^c^Minimum Mg − initial Mg*.

*^d^ΔMg/initial Mg*.

*^e^All grades*.

### Status after Appearance of Hypomagnesemia

**Figure 2:**
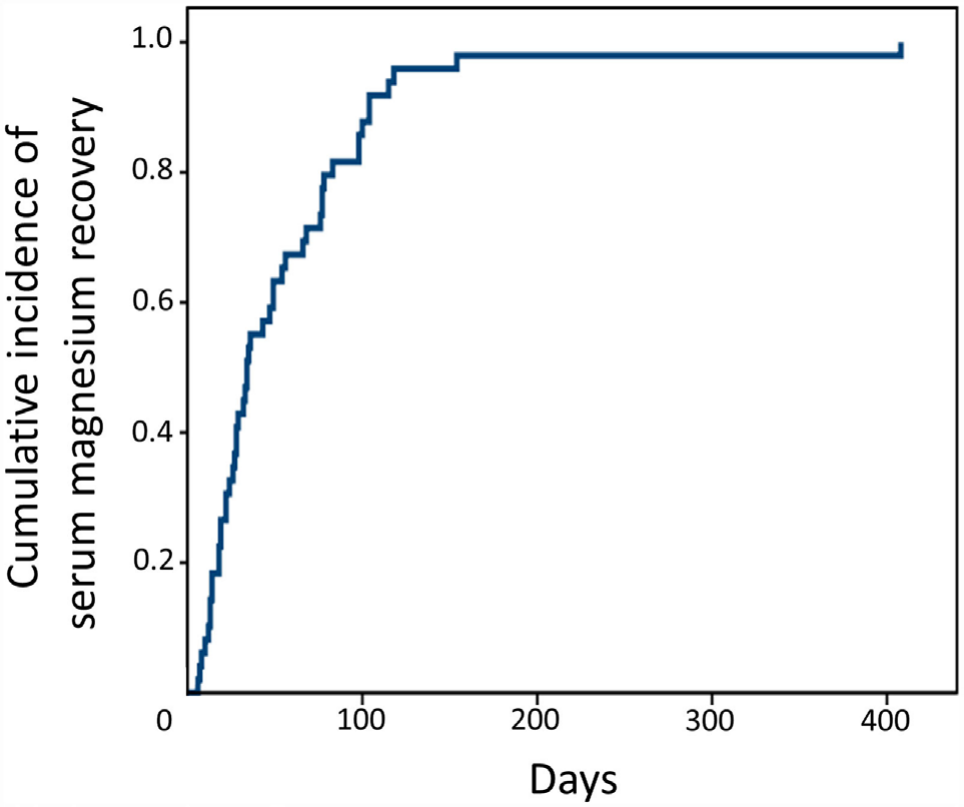
**Time to recovery from hypomagnesemia after stopping Cmab administration**.

Among the 66 patients who developed hypomagnesemia, 61 (94%) continued with Cmab-containing treatment, of whom 52 received intravenous (IV) Mg supplementation. These 61 patients with hypomagnesemia received a cumulative total of 533 Cmab administrations. Intravenous Mg supplementation during treatment was given at 16.6 meq/cycle. Thirteen (25%) of the 52 supplemented patients received oral low-dose Mg sulfate temporarily (median dose 3 g/day). No patient solely discontinued Cmab-containing treatment due to intolerable hypomagnesemia only. In those patients with hypomagnesemia at the time of Cmab discontinuation (*n* = 49), median time to serum Mg recovery to pretreatment levels was 35 days (range 6–408 days), and all patients eventually recovered (Figure 2). Minimum serum Mg level during Cmab-containing treatment, concurrent platinum administration, and number of Cmab administrations were not associated with a longer recovery period (≥35 days).

## Discussion

In this study, we found that Cmab is associated with a significant risk of hypomagnesemia in patients with head and neck cancer who were receiving concurrent platinum therapy for an extended period. Risk varied with treatment form. These findings indicate the importance of early monitoring of serum Mg level during Cmab-based therapy and suggest that although prophylactic Mg supplementation is not necessary, special attention should be given to high-risk patients.

In healthy individuals, serum Mg concentrations are tightly controlled between intestinal absorption and renal excretion and vary between 0.77 mmol/l (1.82 mg/dl) and 1.10 mmol/l (2.33 mg/dl) (9). Eighty percent of serum Mg is filtered in the glomeruli, with 95% being reabsorbed in the nephron. Patients with advanced cancer might develop hypomagnesemia for any of several reasons, besides anti-EGFR therapy, including decreased oral intake, surgery, platinum agents, and diarrhea (10, 11). Hypomagnesemia during anti-EGFR therapy is mainly caused by a decrease in Mg reabsorption in the kidney. The Mg channel transient receptor potential cation channel, sub-family M, member 6 (TRPM6) is involved in the active reabsorption of Mg in the limb of the loop of Henle and distal convoluted tubule. Activation of renal EGFR located at the basolateral membrane is necessary to prevent renal Mg wasting by stimulation of the epithelial Mg channel TRPM6 (12, 13). Tejpar et al. found clear evidence of defective renal Mg handling, namely, an inappropriately high fractional excretion of Mg in the urine in the setting of serum hypomagnesemia (14). In our study, almost all patients showed a progressive decrease in serum Mg concentration, and the overall incidence of hypomagnesemia was comparable with that in a previous report, which mainly examined patients with colorectal cancer (15). However, the incidence of clinically important grade 3/4 hypomagnesemia was relatively low (0.8%) in comparison with a previously reported meta-analysis, in which grade 3/4 hypomagnesemia occurred in 4.8–5.6% of patients receiving Cmab-based therapy (16, 17). The authors suggested that it might be reasonable to start proactive Mg supplement when grade 1 hypomagnesemia occurred. Patients with higher baseline Mg levels tend to have more pronounced Mg wasting, as previously reported (14).

Our multivariate analysis to assess potential variables affecting the development of hypomagnesemia found that the only baseline characteristic, which predicted hypomagnesemia was low baseline Mg level, irrespective of patient age, baseline creatinine clearance, nutrition status, or history of platinum administration. Concurrent platinum administration was also detected as a risk factor for hypomagnesemia. CDDP directly causes cytotoxic damage to the proximal tubule and tubular reabsorption defects, resulting in hypomagnesemia (18). In addition, a recent study showed that CDDP treatment might also downregulate EGF and TRPM6 in the rat kidney, causing renal Mg loss (19). Moreover, CDDP itself has been associated with shifts in erythrocyte cellular-to-plasma Mg ratios (20). Hypomagnesemia is observed in up to 50% of patients treated with CDDP-containing regimens (18, 21). Carboplatin nephrotoxicity is similar in nature to CDDP nephrotoxicity but occurs less frequently and is usually much less severe. Carboplatin also causes tubular damage which leads to hypomagnesemia but is often reversible (22). Mild glomerular impairment and hypomagnesemia have each been reported in up to 25% of children (23–25).

A retrospective study of 114 colorectal cancer patients from the Roswell Park Cancer Institute suggested a direct relationship between the duration of Cmab exposure and hypomagnesemia (26). Tejpar et al. reported similar findings in a prospective study of 98 patients treated with EGFR-targeting monoclonal antibodies with or without chemotherapy (14). Both studies showed that less than 3 months of Cmab exposure was associated with a lower incidence of hypomagnesemia. This finding is important, given that many patients with head and neck cancer receive Cmab for long periods as maintenance. Together, these findings clearly explain the difference in incidence between our present bioradiation group and palliative group. Generally, patients treated with bioradiation receive fewer administrations of Cmab than those receiving palliative chemotherapy and without concurrent platinum agents.

Although an optimal replacement strategy has yet to be determined, Mg replacement should be considered for patients with grade 2 hypomagnesemia with risk factors (elderly, cardiac disease history) and should be offered to patients with grade 3/4 hypomagnesemia from the viewpoint of safety (27). Further, the biologic relationship between Mg and cancer progression is unclear, and there is no definite evidence to suggest that Mg supplementation reverses the anti-tumor effects of EGFR inhibition. This suggests that proactive Mg replacement should be recommended. In our study, after the appearance of hypomagnesemia, we successfully continued Cmab administration mainly under intravenous Mg supplementation, indicating its effectiveness and tolerance. With respect to supplemental method, many patients with metastatic colorectal cancer receiving Cmab developed severe and refractory hypomagnesemia and poorly tolerated oral Mg supplementation due to diarrhea (28). On the other hand, rapid elevation of plasma Mg concentration may inhibit Mg reabsorption, leading to hypermagnesiuria (29, 30). In addition, one-quarter of our present patients who continued Cmab administration after the appearance of hypomagnesemia received oral Mg sulfate in combination with intravenous Mg supplementation without severe diarrhea. The optimum method of Mg supplementation warrants further evaluation. In a previous study (14), hypomagnesemia was reversible and complete recovery was seen once the anti-EGFR targeted agent was discontinued. In our present report, serum Mg levels corrected within 4–6 weeks of stopping Cmab. A stop-and-go approach to Cmab administration is an alternative for patients with severe and refractory hypomagnesemia and without a large tumor burden. Several retrospective studies have examined the role of early hypomagnesemia induced by Cmab as a predictor of efficacy and outcome in colorectal cancer (31, 32). However, the role of hypomagnesemia as predictive of outcome in head and neck patients treated with Cmab has not been clearly established, and we were also unable to assess this because of the heterogeneity of patient characteristics and treatment forms.

Several limitations of the study warrant mention. First, due to a lack of precise information, we did not evaluate other potential variables, such as use of diuretics, diarrhea, or oral intake during treatment. As an example, CDDP treatment also produces gastrointestinal side effects and requires diuretics, which might lead to greater Mg depletion. Second, we did not assess symptoms related to hypomagnesemia with a validated questionnaire. This might have underestimated the symptomatic impact of Cmab-induced hypomagnesemia. Prospective observational studies will provide further evidence to guide practice.

## Conclusion

In this study, we showed that Cmab is associated with a significant risk of hypomagnesemia in patients with head and neck cancer who were receiving longer term administration and concurrent platinum therapy. This risk varies with the treatment form. Early monitoring of serum Mg level is important when Cmab-based therapy is performed, especially in high-risk patients. Evaluation of timing, dose, and route of administration of Mg supplementation, as well as nutritional education, in this population requires further study.

## Author Contributions

TE participated in the study concept and design, interpreted the data, and drafted the manuscript. SS, TY, and TW participated in the study concept and design and interpreted the data. MT extracted, managed, and analyzed the data. All authors provided critical revisions and approved the final manuscript.

## Conflict of Interest Statement

MT receives honoraria from Merck Serono. The remaining co-authors declare that the research was conducted in the absence of any commercial or financial relationships that could be construed as a potential conflict of interest. The reviewer NK declared a past co-authorship with one of the authors (MT) to the handling Editor, who ensured that the process met the standards of a fair and objective review.
